# Lymphovascular invasion have a similar prognostic value as lymph node involvement in patients undergoing radical cystectomy with urothelial carcinoma

**DOI:** 10.1038/s41598-018-34299-6

**Published:** 2018-10-29

**Authors:** Hyeong Dong Yuk, Chang Wook Jeong, Cheol Kwak, Hyeon Hoe Kim, Ja Hyeon Ku

**Affiliations:** 1 Department of Urology, Inje University Sanggye Paik Hospital, Seoul, Korea; 2 Department of Urology, Seoul National University Hospital, Seoul, Korea

## Abstract

To determine the prognostic value of lymphovascular invasion (LVI) in patients with bladder cancer who underwent radical cystectomy. Total of 747 patients underwent radical cystectomy; of these, only 164 did not undergo lymph node dissection (LND). The patients were divided into 4 groups: N0, N1, LVI without LND, and non-LVI without LND. Patients in the N1 and LVI groups had significantly higher T stages and grades, as well 1.5- to 2-fold higher recurrence and mortality rates. Overall survival (OS) was significantly poorer in the N1 group, compared with the N0 and non-LVI groups (p = 0.001 and 0.012), and in the LVI group relative to the N0 and non-LVI groups (p = < 0.001 and <0.001). Recurrence-free survival (RFS) was also significantly poorer in the N1 group relative to the N0 and non-LVI groups (p = < 0.001 and <0.001), and in the LVI group relative to the N0 and non-LVI groups (p = < 0.001 and <0.001). Among patients undergoing radical cystectomy, the clinical results predicted by LVI were similar to those predicted by lymph node involvement. Therefore, the role of adjuvant chemotherapy or immunotherapy may need to be prospectively evaluated in LVI-positive patients regardless of T stage after radical cystectomy.

## Introduction

Bladder cancer is among the most common malignancies of the urogenital system^1^, and most cases are histopathologically identified as urothelial carcinoma (UC). Among patients with bladder UC, 75% present with non-muscle-invasive bladder cancer (NMIBC) at the time of diagnosis, 70% will experience a recurrence within 1 year and 20–30% will progress to muscle-invasive bladder cancer (MIBC)^2–4^. MIBC is associated with a poor prognosis and is the 13th most common cause of mortality worldwide, and its incidence is gradually increasing with population aging^5,6^.

A variety of treatment options for UC are available, including surgery, intravesical Bacille Calmette-Guerin (BCG) therapy, neoadjuvant/adjuvant chemotherapy, immunotherapy, and multimodal therapy. The selection of an appropriate treatment with respect to the patient and disease characteristics is prognostically important, and accordingly the tumor stage and grade are important criteria for such treatment decisions. However, several other factors can contribute to a treatment decision, including lymphovascular invasion (LVI). LVI, defined as the presence of tumor cells in the lymphatic vessels and vascular wall, can affect the characteristics of UC and may be predictive of the outcome^7^. In this study, we analyzed differences in prognosis according to the presence or absence of LVI after radical cystectomy without lymph node dissection (LND), which is a rare event, and discussed the clinical and prognostic value of LVI.

## Methods

### Study sample

The Institutional Review Board at our center approved this retrospective study (H-1707-067-869) and waived the requirement to obtain informed consent from the patients. All research and related protocols used in this study complied with the principles of the Declaration of Helsinki.

We retrospectively reviewed the medical records of 857 patients with N1 or lower UC who underwent radical cystectomy at Seoul National University Hospital from 1991 to 2016, including patients who did not undergo LND. After excluding cases for which the pathologic finding was not UC, 747 patients were included in the analysis.

### Study design

We divided the included patients into four groups: N0, N1, non-LVI without LND and LVI without LND. Patients who underwent LND were divided into N0 and N1 groups according to N stage, whereas patients who did not undergo LND were divided into non-LVI and LVI groups according to LVI status.

For diagnostic accuracy, the extracted specimens were reviewed by two experienced genitourinary pathologists. The TNM stage and tumor grade were determined according to the 2010 American Joint Committee on Cancer classification and the 2004 World Health Organization/International Society of Urologic Pathology consensus classifications. We additionally collected clinicopathological information such as age, height, weight, body mass index (BMI), sex, American Society of Anesthesiologists (ASA) physical status, pathologic TNM stage, carcinoma *in situ* (CIS) status, LVI, margin-positive status, LND enforcement, number of removed lymph nodes, number of positive lymph nodes, and neoadjuvant chemotherapy enforcement. We also collected various oncological data, including the initial recurrence and mortality.

In the absence of any recurrence or metastasis, most patients were followed up every 3 months for 3 years after radical cystectomy, every 6 months during years 4 and 5, and annually thereafter. Each follow-up included routine laboratory and urine testing and urine cytology. Neo-bladder patients also underwent an ultrasonic bladder scan for a residual urine check. Radiological examinations such as computed tomography (CT) and bone scans were performed annually.

### Statistical analysis

Regarding the statistical analysis, continuous variables are presented as mean values with standard deviations (SDs) or as median values with interquartile ranges (IQRs). Categorical variables are presented as frequencies of events (%). The primary and secondary endpoints of the study were overall survival (OS) and recurrence-free survival (RFS), respectively, and all survival outcomes were estimated using the Kaplan–Meier method and compared using the log-rank test. A Cox proportional hazard regression analysis was used to identify the predictors of various oncological outcomes. All statistical tests were performed using IBM SPSS Statistics, version 22.0 (IBM, Armonk, NY, USA), and a p-value of <0.05 was considered to indicate statistical significance.

## Results

### Clinicopathological characteristics of the patients

Table 1 lists the clinicopathological characteristics and oncologic results of the 747 patients included in this study during a median follow-up duration of 41.5 (IQR: 6–311) months. The median age of the patients was 66.0 (IQR: 21–95), and >80% of the subjects were male. The four groups differed with respect to T stage, tumor grade and CIS status. The N1 and LVI groups had a higher frequency of stage T2 and higher cases and a higher tumor grade, compared with the other groups (p = < 0.001 and <0.001, respectively), whereas the LVI group had a significantly lower frequency of CIS cases relative to the other groups (p = 0.007). Additionally, the N1 and LVI groups had significantly higher recurrence and mortality rates, compared with the other groups (p = < 0.001 and <0.001, respectively).

**Table 1:** Clinicopathological characteristics of the study patients.

Parameter	N0	N1	Non-LND with LVI	Non-LND without LVI	P-value
Number	536	47	58	106	
Mean Age	64.8 ± 10.1	63.3 ± 10.6	61.6 ± 13.0	64.6 ± 9.6	
BMI	22.94 ± 3.14	22.91 ± 2.44	23.42 ± 3.85	23.67 ± 2.92	
Sex					0.542
Male	447 (83.4%)	38 (80.9%)	52 (89.7%)	91 (85.8%)	
Female	89 (16.6%)	9 (19.1%)	6 (10.3%)	15 (14.2%)	
ASA					0.112
0	2 (0.4%)	0 (0%)	0 (0%)	1 (0.9%)	
1	174 (32.6%)	21 (44.7%)	21 (36.2%)	44 (41.5%)	
2	327 (61.2%)	25 (53.2%)	32 (55.2%)	49 (46.2%)	
3	31 (5.8%)	1 (2.1%)	5 (8.6%)	12 (11.3%)	
T stage					<0.001
T0	104 (19.4%)	4 (8.5%)	1 (1.7%)	15 (14.2%)	
Ta	29 (5.4%)	0 (0%)	0 (0%)	7 (6.6%)	
Tis	64 (11.9%)	0 (0%)	1 (1.7%)	14(13.2%)	
T1	107 (20.0%)	3 (6.4%)	2 (3.4%)	32 (30.2%)	
T2	103 (19.2%)	13 (27.7%)	21 (36.2%)	25 (23.6%)	
T3	117 (21.8%)	22 (46.8%)	31 (53.4%)	11 (10.4%)	
T4	12 (2.2%)	5 (10.6%)	2 (3.4%)	2 (1.9%)	
Grade					<0.001
Unknown	92 (17.2%)	3 (6.4%)	1 (1.7%)	27 (25.5%)	
LG	20 (3.7%)	0 (0%)	3 (5.2%)	12 (11.3%)	
HG	424 (79.1%)	44 (93.6%)	54 (93.1%)	67 (63.2%)	
CIS	181 (33.8%)	14 (29.8%)	8 (13.8%)	26 (24.5%)	0.007
Margin positive	9 (1.7%)	2 (4.3%)	1 (1.7%)	0 (0%)	0.288
Removed LN number	17.44 ± 10.9	16.0 ± 11.5	0	0	
Positive LN number	0.2 ± 0.2	0.9 ± 0.1	0	0	
Neoadjuvant Chemotherapy	69 (12.9%)	7 (14.9%)	8 (13.8%)	16(15.1%)	0.921
Recurrence	146 (27.2%)	25 (53.2%)	35 (60.3%)	31 (29.2%)	<0.001
Mortality	173 (32.3%)	25 (53.2%)	46 (79.3%)	55 (51.9%)	<0.001

LVI, lymphovascular invasion; LND, lymph node dissection; BMI, body mass index; ASA, American Society of Anesthesiologists score; Tis, tumor *in situ*; LG, low-grade; HG, high-grade; CIS, carcinoma *in situ*; LN, lymph node.

### Inter-group comparison of the association of LVI with OS

**Figure 1:**
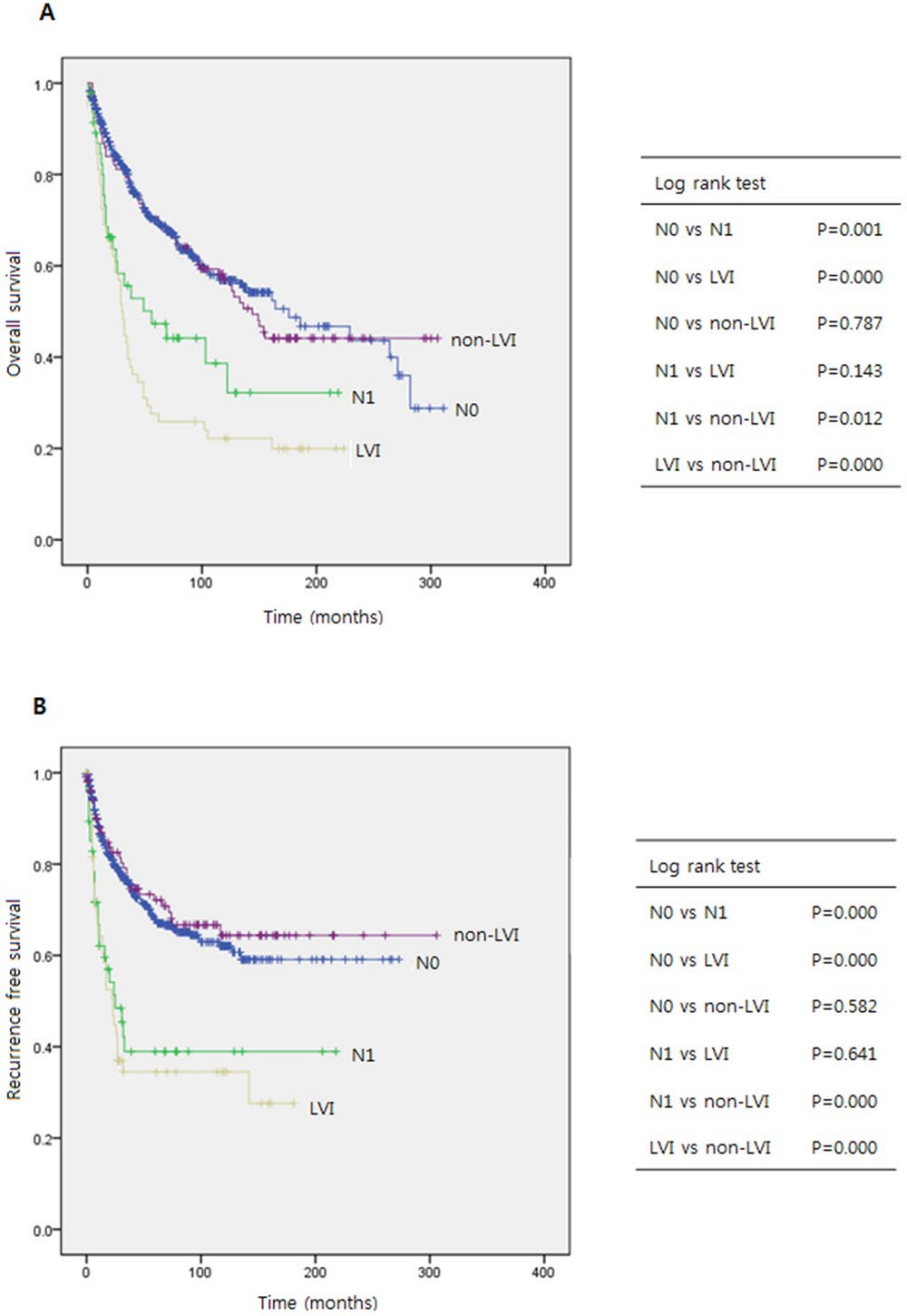
Overall and recurrence-free survival analyses. Kaplan-Meier survival curves and Log rank test for comparing (**A**) overall and (**B**) recurrence free suvivals according to the 4 groups: N0, N1, LVI without LND, and non-LVI without LND.

Figure 1A presents a graph of OS in each of the groups. OS was relatively better in the non-LVI and N0 groups, compared with the LVI and N1 groups. A log-rank comparison confirmed that the N0 and non-LVI groups had significantly better OS outcomes, when compared with the N1 and LVI groups (p = 0.001 and <0.001, and p = 0.012 and <0.001, respectively). However, no significant differences in OS were observed between the N0 and non-LVI groups, or between the N1 and LVI groups.

### Intergroup comparison of the association of LVI with RFS

Figure 1B presents a graph of RFS in each of the groups. The results obtained for RFS are similar to those for OS. Specifically, the N0 and non-LVI groups appeared to have better RFS outcomes when compared with the N1 and LVI groups, and a log-rank test confirmed both groups achieved a significantly better RFS than did the N1 and LVI groups (p = < 0.001 and <0.001, and p = < 0.001 and <0.001, respectively). Again, no significant differences in RFS were observed between the N0 and non-LVI groups or between the N1 and LVI groups.

### Multivariate regression analysis of significant predictors of OS and RFS in the overall study population

We also performed a multivariate Cox regression analysis to identify significant predictors of OS and RFS after radical cystectomy. Regarding OS, age (hazard ratio [HR]: 1.05; 95% confidence interval [CI]: 1.03–1.06), LVI (HR: 2.50; 95% CI: 1.68–3.70), stage N1 disease (HR: 1.72; 95% CI: 1.11–2.56) and stage T3 (HR: 3.37; 95% CI: 1.30–5.54) or T4 disease (HR: 4.92; 95% CI: 1.38–8.18) were identified as significant predictors. These parameters were also identified as significant predictors of RFS (age, HR: 1.02, 95% CI: 1.00–1.03; LVI, HR: 2.22, 95% CI: 0.343–3.685 N1, HR: 2.07, 95% CI: 1.20–3.58; T3, HR: 3.03, 95% CI: 1.41–6.50 and T4, HR: 4.25, 95% CI: 1.65–10.92) (Table 2).

**Table 2:** Multivariable Cox regression analysis of the overall population.

Parameter	Overall survival		Recurrence-free survival	
	HR (95% CI)	P-value	HR (95% CI)	P-value
Age	1.050 (1.037–1.063)	<0.001	1.020 (1.007–1.033)	0.003
T stage		<0.001		0.002
T0	reference		reference	
Tis	0.913 (0.505–1.650)	0.764	1.899 (0.922–3.914)	0.082
Ta	1.441 (0.593–3.499)	0.420	1.957 (0.756–5.066)	0.166
T1	1.373 (0.656–2.874)	0.400	1.875 (0.866–4.061)	0.111
T2	1.187 (0.569–2.477)	0.648	1.684 (0.779–3.638)	0.185
T3	3.373 (1.306–5.545)	0.007	3.032 (1.413–6.507)	0.004
T4	4.926 (1.389–8.189)	0.007	4.258 (1.659–10.926)	0.003
N stage & LVI		<0.001		0.001
N0	reference		reference	
N1	1.722 (1.115–2.658)	0.014	2.003 (1.286–3.118)	0.002
Non-LVI	1.085 (0.781–1.507)	0.375	1.074 (0.713–1.617)	0.733
LVI	2.502 (1.689–3.707)	<0.001	2.019 (1.309–3.114)	0.001

HR, hazard ratio; CI, confidence interval; LVI, lymphovascular invasion.

### Significant predictors of OS and RFS in the Nx subgroup population

We finally performed a Cox regression analysis to identify the significant predictors of oncological outcomes (e.g., OS and RFS) in the Nx subgroup without LND. Here, age (HR: 1.04; 95% CI: 1.02–1.06) and LVI (HR: 2.42; 95% CI: 1.51–3.90) were identified as predictors of OS, whereas LVI (HR: 2.04; 95% CI: 0.14–3.66) was the only identified predictor of RFS (Table 3).

**Table 3:** Multivariable Cox regression analysis of the Nx subgroup.

Parameter	Overall survival		Recurrence free survival	
	HR (95% CI)	P-value	HR (95% CI)	P-value
Age	1.042 (1.020–1.064)	<0.001	1.009 (0.986–1.032)	0.451
T stage		0.651		0.479
T0	reference		reference	
Tis	1.093 (0.264–2.799)	0.803	0.325 (0.034–3.131)	0.330
Ta	1.178 (0.273–4.377)	0.900	0.324 (0.022–4.879)	0.416
T1	1.780 (0.277–5.005)	0.825	0.591 (0.069–5.036)	0.630
T2	0.848 (0.424–7.473)	0.431	0.649 (0.077–5.490)	0.692
T3	0.774 (0.120–6.001)	0.869	1.071 (0.127–9.057)	0.950
T4	0.860 (0.170–3.522)	0.741	0.401 (0.022–7.222)	0.535
Grade		0.837		0.231
Unknown	reference		reference	
Low	0.774 (0.202–2.966)	0.708	1.737 (0.194–15.519)	0.521
High	0.996 (0.294–3.374)	0.996	3.927 (0.513–30.090)	0.188
LVI	2.428 (1.511–3.903)	<0.001	2.048 (0.146–3.661)	0.016

H. R., hazard ratio; C. I., confidence interval; LVI, lymphovascular invasion.

## Discussion

Whereas previous studies focused on the prognosis and significance of LVI in any subgroup, our study analyzed the prognostic risk of N0 and N1 in the presence of LVI. Accordingly, we analyzed differences in oncological prognosis according to the presence or absence of LVI in the patients who underwent radical cystectomy without LND, as well as differences among pathologically confirmed N0 and N1 patients who underwent radical cystectomy with LND. Furthermore, we evaluated the effects of LVI on the oncological prognosis with respect to lymph node invasion by comparing patients with LVI in the Nx group to those in the N0 and N1 groups. Here, we did not identify the exact difference in oncological prognosis between the LVI and N1 groups and the survival curves of the two groups were similar, with both exhibiting significantly poorer RFS and OS outcomes relative to the N0 group. Similarly, the non-LVI group, which did not differ significantly from the N0 group, had better RFS and OS outcomes when compared with the N1 group. Furthermore, our overall population multivariate Cox regression analysis identified N1, LVI, T stage and age as significant predictors of the oncological prognosis of patients undergoing radical cystectomy. However, in the Nx subgroup multivariate Cox regression analysis, LVI was the only significant predictor of oncological prognosis.

LVI, defined as the presence of tumor cells in small vessels. This process increases the frequency of lymph node metastasis and is an important step in the processes of metastatic spread and cancer cell microcirculation, as^8–10^ cancer cells migrate through the local lymphatics after invading and proliferating the lymphovascular space^11^. LVI is observed in many types of cancer, and has been identified as an important predictor of poor prognosis in patients with liver, testicular and penile cancer^12,13^. By contrast, the reported outcomes of LVI in patients with bladder cancer are varied, although the positive outcomes are not yet generally accepted^14–19^.

Previous studies have identified LVI as an independent predictor of OS, CSS and RFS in patients who have undergone radical cystectomy. Bolenz *et al*. determined that LVI is a significant predictor of OS and RFS in node-negative patients, and that the tumor stage and grade are significant predictors^20^. In a comparison of node-positive and -negative subgroups, Shariat *et al*. found that LVI correlated with OS, CSS and RFS in all subgroups and observed that the tumor stage and LN involvement were risk factors for survival^14^. Hermann *et al*. also reported that LVI, tumor stage and LN status were important prognostic factors for OS^21^. However, some studies have reported that LVI only affects the prognosis of patients with N0 disease, and not all radical cystectomy patients. Lotan *et al*. reported that LVI is an important risk factor for local, distant and overall recurrence in node-negative patients, but not in node-positive patients^22^. Tilki *et al*. reported that LVI is associated with recurrence and survival only in patients with pT1N0 disease. Still other studies observed no significant correlation between LVI and survival^23,24^.

We note that our current research had some limitations. First, this study featured a retrospective design, and the surgeries were performed by four surgeons. Second, although we considered neoadjuvant chemotherapy, we did not consider previous repeated transurethral resection of bladder tumor (TURBT) or intravesical treatment, although these may have affected the outcomes. Third, there may be potential interoperability variability or limitations in setting up a single center. Fourth, the small sample size of patients who did not undergo LND. Fifth, there is variability in follow-up and at the time of test. Sixth, when cystectomy is performed in UC, LND is performed with cystectomy as standard treatment. The absence of LND can be a confounding bias. Seventh, regimens and time from neoadjuvant chemotherapy completion to surgery was not consistent

## Conclusion

LVI is an independent predictor of prognosis in patients with bladder UC who have undergone radical cystectomy. Relatively speaking, LVI and N1 are prognostically similar, and both are associated with a poorer prognosis when compared with N0. Therefore, the role of adjuvant chemotherapy or immunotherapy may need to be prospectively evaluated in LVI-positive patients regardless of T stage after radical cystectomy.
